# Aortic Endograft Infection After COVID-19: A Single-Centre Case Series and a Proposed Descriptive Clinical–Radiological PET/CT Staging Framework

**DOI:** 10.3390/diagnostics16203355

**Published:** 2026-10-15

**Authors:** Sherif Sultan, Kalliopi-Maria Tasopoulou, Yogesh Acharya, Henri Bartolozzi, Nicole Gallagher, William Wijns, Osama Soliman

**Affiliations:** 1 Department of Vascular and Endovascular Surgery, University Hospital Galway, University of Galway, H91YR71 Galway, Ireland; 2 Western Vascular Institute, Department of Vascular Surgery and Endovascular Surgery, Galway Clinic, Doughiska, Royal College of Surgeons in Ireland and University of Galway, Galway Affiliated Hospital, H91 HHT0 Galway, Ireland; 3 Department of Medicine, Royal College of Surgeons in Ireland (RCSI), 123 St Stephen’s Green, D02 YN77 Dublin, Ireland; 4 Department of General Practice, Letterkenny University Hospital, Letterkenny, Donegal F92 AE81 & Irish College of GPs, 15 Hogan Place, 2D02 DK23 Dublin, Ireland; 5 The Euro Heart Foundation, 2501 CH Den Haag, The Netherlands; 6 Department of Cardiovascular Medicine, Precision Cardiovascular Medicine & Innovation Institute (PCMI), Cardiovascular Research Institute Dublin (CVRI Dublin), Mater Private Network & RCSI University of Medicine and Health Sciences, Eccles Street, D07KWR1 Dublin, Ireland

**Keywords:** aortic endograft infection, EVAR, COVID-19, PET/CT, FDG, MAGIC criteria, case series

## Abstract

**Background**: Aortic endograft infection (AEI) after endovascular aneurysm repair (EVAR) is rare but life-threatening. The Management of Aortic Graft Infection Collaboration (MAGIC) criteria define infection, but there is no standard way to report the extent of infection. We describe patients who developed AEI after coronavirus disease 2019 (COVID-19) and propose a descriptive clinical–radiological PET/CT staging framework. **Methods**: Retrospective, single-centre case series of EVAR patients who underwent [18F] fluorodeoxyglucose positron emission tomography/computed tomography (FDG PET/CT) between 2020 and 2023. Six index patients (Group A) were managed for AEI after PCR-confirmed COVID-19. Five reference patients without COVID-19, imaged during the same period for suspected infection at another site (Group B, *n* = 2) or malignancy (Group C, *n* = 3), were included to describe endograft uptake in the absence of AEI; they were not matched controls. Each patient was classified according to MAGIC, with the finding supporting each criterion recorded. Each endograft was assigned a descriptive clinical–radiological stage (0–III): Stages 0–II were defined by PET/CT uptake pattern and anatomical extent, and Stage III by an additional imaging-defined (e.g., endograft limb thrombosis) or clinical (sepsis) complication, recorded separately. **Results**: Mean age was 76 years (9 men). Of the six index patients, one (Case 6, organism cultured from the explanted endograft) met the MAGIC definition of diagnosed AEI and five met that of suspected AEI. All six presented with fever, and every index patient had at least one additional source of bacteraemia or cause of immunosuppression, including corticosteroids given for COVID-19 pneumonia. Five had iliopsoas abscess. Four index endografts were classified as Stage III (clinical complication, sepsis, in all four; imaging-defined complication, endograft limb thrombosis, also in one) and two as Stage II; all five reference endografts were Stage 0. The median interval from COVID-19 to AEI diagnosis was 14 months (IQR 10–16), and from EVAR to PET/CT 15 months (IQR 4–35). Three index patients died (two infection- or aorta-related), and all three Group C patients died of malignancy progression. **Conclusions**: This case series describes late AEI, frequently with iliopsoas involvement, in patients with prior COVID-19 and multiple other risk factors. The design does not permit any conclusion as to whether COVID-19 contributes to endograft infection. The proposed descriptive clinical–radiological PET/CT framework may help standardise the reporting of disease extent alongside MAGIC-based diagnosis but requires validation in larger, independent cohorts before any diagnostic or therapeutic use.

## 1. Introduction

Aortic endograft infection (AEI) is a potentially fatal complication following endovascular aneurysm repair (EVAR). Approximately 0.6% of EVAR endografts become infected, with a reported range of 0.4% to 0.8% [1]. However, the actual incidence may be underreported in the literature, as diagnosing AEI is challenging due to the heterogeneous clinical manifestations and radiological findings [2]. Furthermore, the aetiology of AEI is multifactorial, as several risk factors can result in exposure of the endograft to infection, including breaches of aseptic technique, adjacent infections, and bacteraemia [3]. Similarly, patient-related factors, like malignancy, chemotherapy, malnutrition, immune disorders, diabetes mellitus, liver and renal function impairment, or corticosteroid administration, could also be implicated in AEI [3]. Additionally, viral infections such as HIV (human immunodeficiency virus) are known to compromise the capacity of the immune system [4,5,6].

Over the past five years, following the coronavirus disease 2019 (COVID-19) pandemic, considerable research has focused on the systemic and long-term effects of severe acute respiratory syndrome coronavirus 2 (SARS-CoV-2) infection, which can lead to overstimulation of the immune response followed by immune exhaustion and immunosuppression [7]. Whether prior SARS-CoV-2 infection has any bearing on subsequent EVAR graft infection has received little attention. In this retrospective case series, we describe six patients under EVAR surveillance at our institution who were managed for AEI after documented COVID-19 during 2020–2023. To illustrate PET/CT appearances of endografts without infection, we also describe five EVAR patients without COVID-19 who underwent PET/CT during the same period for other indications. The aims of the study are (i) to describe the clinical course, microbiology, PET/CT appearances and management of these patients, and (ii) to propose, for future validation, a descriptive clinical–radiological PET/CT framework for reporting the anatomical extent and complications of endograft infection that complements MAGIC-based diagnosis [8,9]. The study was not designed to assess whether COVID-19 is associated with, or causes, endograft infection.

## 2. Methods

**Study design, setting and case identification**: 

This is a retrospective, single-centre descriptive case series from the Western Vascular Institute (University Hospital Galway and Galway Clinic), a tertiary vascular referral centre. The source population comprised patients who had undergone EVAR for infrarenal abdominal aortic aneurysm (AAA) and who remained under institutional imaging surveillance. Patients were eligible if they had (i) EVAR for infrarenal AAA, (ii) at least one fluorodeoxyglucose ([18F]FDG) PET/CT covering the endograft between 2020 and 2023, during the COVID-19 pandemic, and (iii) complete clinical, microbiological and imaging records.

Index cases were the patients evaluated and treated at our institution for suspected or diagnosed aortic endograft infection during this period (n = 6); all had PCR-confirmed COVID-19 before the diagnosis of AEI. Reference cases were EVAR patients without documented COVID-19 who underwent PET/CT at our institution during the same pandemic period for another clinical indication—suspected infection at another site (n = 2) or staging of malignancy (n = 3)—and in whom AEI was not diagnosed (n = 5).

Given the observational nature of the study and the use of existing patient records, no direct patient or public engagement was undertaken.

### 2.1. Index and Reference Cases

- **Group A—index cases (n = 6):** EVAR patients with PCR-confirmed COVID-19 who were subsequently evaluated and treated for AEI, classified by MAGIC as diagnosed or suspected (Table 1), each with at least one additional documented source of infection or cause of immunosuppression.

**Table 1:** MAGIC criteria met by each patient, with the supporting finding, its timing after EVAR and potential alternative sources.

Case	Clinical Criteria	Radiological Criteria (Months After EVAR)	Laboratory Criteria	Potential Alternative Source or Route	MAGIC Classification
**Group A (index cases)**					
Case 1	Minor: fever ≥ 38 °C, AEI judged the most likely cause	Minor: focal FDG uptake in aneurysm sac (SUVmax 6) and left iliac limb (SUVmax 16) on PET/CT; left iliac limb thrombosis on CT; no collection or gas (14)	Minor: *Staphylococcus aureus* in blood culture, no apparent source other than AEI	Shoulder dislocation and reduction (no infective focus)	Suspected (3 minor criteria, 3 categories)
Case 2	Minor: fever ≥ 38 °C with weight loss, AEI judged the most likely cause by the MDT	Minor: focal FDG uptake in endograft and aneurysm sac (SUVmax 11) with contiguous left iliopsoas abscess (SUVmax 24) and left ureteric involvement (67)	Not met: blood cultures negative	Left thigh cellulitis	Suspected (2 minor criteria, 2 categories)
Case 3	Minor: fever ≥ 38 °C with abdominal pain, AEI judged the most likely cause	Minor: FDG uptake in aneurysm sac (SUVmax 6) with iliopsoas abscess on CT, percutaneously drained (20)	Minor: *Staphylococcus capitis* in blood culture	Translumbar embolization of type II endoleak (route of sac access rather than an independent source)	Suspected (3 minor criteria, 3 categories)
Case 4	Fever ≥ 38 °C present: not scored (concurrent *Salmonella* UTI)	Major: perigraft collection contiguous with the endograft on CT, ≥3 months after EVAR. Minor: intense FDG uptake in aneurysm sac (SUVmax 12.4) and FDG-avid left iliopsoas abscess (SUVmax 20.1) (4)	*Salmonella enteritidis* in blood culture: not scored (same organism isolated in urine)	*Salmonella* UTI; lung adenocarcinoma	Suspected (1 major radiological criterion); revised from diagnosed
Case 5	Minor: fever ≥ 38 °C, AEI judged the most likely cause	Minor: circumferential FDG uptake in aneurysm sac (SUVmax 6.8) with left iliopsoas abscess on CT (9)	Minor: *Corynebacterium jeikeium*, *Bacteroides fragilis* and *Candida glabrata* in blood cultures	CT-guided retroperitoneal lymph node biopsy; aortic cuff and coil embolization for type Ia endoleak (routes of access)	Suspected (3 minor criteria, 3 categories)
Case 6	Minor: fever ≥ 38 °C with lower back pain	Minor: marked FDG uptake in endograft and iliac limbs (SUVmax 10) with left iliopsoas abscess (SUVmax 11) (24)	Major: *Escherichia coli* cultured from the explanted endograft (blood cultures negative)	Infected cardiac pacemaker; multiple myeloma	Diagnosed (1 major laboratory and 2 minor criteria)
**Group B (reference cases)**					
Case 7	Rigours, fatigue (attributed to UTI)	No MAGIC criterion; diffuse, homogeneous low-grade endograft uptake, Stage 0 (16)	*Escherichia coli* in urine; blood cultures negative	*E. coli* UTI	AEI not suspected
Case 8	Abdominal pain, lethargy	Endograft: no MAGIC criterion (4). Femorofemoral bypass: 1 major and 1 minor radiological criterion (perigraft collection)	Blood cultures negative	Femorofemoral bypass graft collection	AEI not suspected; bypass graft infection suspected (outside the scope of endograft staging)
**Group C (reference cases)**					
Case 9	—	No MAGIC criterion; no endograft uptake (0.7)	Not applicable	Lung adenocarcinoma	AEI not suspected
Case 10	—	No MAGIC criterion; diffuse, homogeneous low-grade endograft uptake, Stage 0 (78)	Not applicable	Non-Hodgkin lymphoma	AEI not suspected
Case 11	—	No MAGIC criterion; no endograft uptake (N/A)	Not applicable	Lung adenocarcinoma	AEI not suspected

MAGIC diagnosed: one major criterion plus any criterion from a different category; suspected: a single major criterion, or two or more minor criteria from different categories [8]. Minor criteria that MAGIC conditions on the absence of an apparent alternative source were not scored when the same organism was isolated from a concurrent infection at another site (Case 4). Months after EVAR, refer to the CT or PET/CT at diagnosis of AEI (index cases) or to PET/CT (reference cases). No patient had perigraft gas on CT. AEI: aortic endograft infection; CT: computed tomography; EVAR: endovascular aneurysm repair; FDG: fluorodeoxyglucose; MAGIC: Management of Aortic Graft Infection Collaboration; MDT: multidisciplinary team; N/A: not available; PET/CT: positron emission tomography/computed tomography; SUVmax: maximum standardised uptake value; UTI: urinary tract infection.

- **Group B—reference cases (n = 2):** EVAR patients without documented COVID-19 who underwent PET/CT for suspected infection at another site (urinary tract infection; collection around a femorofemoral bypass graft), in whom AEI was not diagnosed.
- **Group C—reference cases (n = 3):** EVAR patients without documented COVID-19 who underwent PET/CT for staging of malignancy, with no clinical suspicion of AEI.

Reference cases were neither matched to index cases nor randomly selected. They are included solely to describe the PET/CT appearance of endografts without infection, and no statistical comparison between groups was undertaken.

All Group A patients had PCR-confirmed SARS-CoV-2 infection before the diagnosis of AEI, with cycle threshold (Ct) values of 20–30. The most common symptoms of COVID-19 were fever, dyspnoea, cough, malaise and lethargy, and all six received corticosteroids for COVID-19 pneumonia in accordance with Health Service Executive (HSE) guidance. Clinical suspicion of AEI arose from persistent or recurrent fever (all six patients), malaise or lethargy (83%), and localised back, flank or groin pain (67%). Laboratory findings included leukocytosis (mean white blood cell (WBC) count > 12 × 10^9^/L) and elevated C-reactive protein (CRP > 100 mg/L). CT angiography (CTA) findings, with their timing after EVAR, are given for each patient in Table 1: five patients (Cases 2–6) had an iliopsoas abscess and/or perigraft collection, Case 1 had thrombosis of the left iliac limb of the endograft, and no patient had perigraft gas. The median interval between COVID-19 and the diagnosis of AEI was 14 months (interquartile range (IQR) 10–16), and the median interval between EVAR and PET/CT imaging was 15 months (IQR 4–35); the interval from EVAR to diagnosis or PET/CT, follow-up and subsequent course are given for each patient in Table 2. Median follow-up after PET/CT for the whole series was 342 days (range 23–1445).

Clinical data collected included demographics, comorbidities, type of EVAR procedure (elective/urgent), aneurysm diameter, presence of post-implantation syndrome, pre- and post-operative blood tests (WBC, CRP), type of endograft (polytetrafluoroethylene (PTFE) or polyester-based), type of vascular access, time from EVAR to PET/CT, microbiology (organism and specimen source), PET/CT findings and patient outcomes. Data are presented descriptively: continuous variables as mean ± standard deviation or median (IQR) according to the Shapiro–Wilk test, and categorical variables as frequencies and percentages. No inferential statistical comparisons were performed. IBM SPSS v29.0.2.0 (IBM Corporation, Armonk, NY, USA) was used for data analysis.

### 2.2. PET/CT Acquisition and Image Analysis

**Acquisition.** All studies were performed on a Philips Gemini TF PET/CT system (Philips Healthcare, Best, The Netherlands) [10] at the Galway Clinic, according to the institutional clinical protocol, which follows the EANM/SNMMI procedure standard for [18F]FDG PET in infection and inflammation [11]. Patients fasted for 6 h before the examination, with only plain water permitted, and blood glucose was measured before injection; [18F]FDG was administered only when blood glucose was below 11 mmol/L [11]. A weight-based activity of 3–5 MBq/kg [18F]FDG was injected intravenously, after which patients rested in an uptake room, and imaging commenced at least 60 min after injection [11]. A low-dose CT from skull base to mid-thigh was acquired for attenuation correction and anatomical localisation, followed by a three-dimensional PET acquisition over the same range (total acquisition time up to 30 min). PET images were reconstructed iteratively with CT-based attenuation correction and reviewed as axial, coronal and sagittal PET, CT and fused PET/CT images. Study-level reconstruction settings (number of iterations and subsets, matrix and voxel size, post-reconstruction filter), CT exposure settings, and patient-level injected activity, uptake time and blood glucose were not retrievable from the retrospective records; they are therefore not reported and are acknowledged as a limitation.

**Image analysis.** In addition to the formal clinical report, each study was reviewed independently by two experienced vascular interventionalists. For each endograft, they recorded (i) the uptake pattern (none; diffuse and homogeneous; focal or heterogeneous); (ii) the anatomical extent (endograft, aneurysm sac, peri-aortic tissue, iliopsoas muscle, ureter/renal pelvis, perigraft collection); and (iii) the SUVmax of the endograft, aneurysm sac and any adjacent collection, measured over the area of most intense uptake. Uptake intensity was assessed visually against the mediastinal blood pool and liver. Each endograft was then assigned a descriptive stage (Table 3), and a consensus stage was agreed where readings differed. Because SUV and target-to-background ratios have not been validated for distinguishing infection from inflammation [11], and the ESVS 2026 guidelines state that SUVmax cut-offs are unreliable owing to overlap between infected and non-infected devices [9], SUVmax is reported descriptively and was not used to define stage.

### 2.3. Diagnostic Classification (MAGIC Criteria)

Each patient was classified according to the MAGIC case definition [8], which remains the recommended case definition for vascular graft and endograft infection in the European Society for Vascular Surgery (ESVS) 2026 guidelines (Recommendation 1, Class I, Level C) [9]. The major and minor criteria were extracted from the source records, together with the date of each finding and the source of each microbiological specimen (Table 1). In accordance with MAGIC, infection was classified as diagnosed (one major criterion plus any criterion from a different category) or suspected (a single major criterion, or two or more minor criteria from different categories) [8]; when infection is suspected, ESVS 2026 recommends further clinical, radiological and laboratory assessment (Recommendation 2, Class I, Level C) [9]. Because the major laboratory criteria depend on explanted, intra-operative or radiologically guided perigraft specimens, patients managed without surgery may remain in the suspected category [12]. Minor criteria that MAGIC conditions on the absence of an apparent alternative source (fever with AEI as the most likely cause; positive blood cultures or raised inflammatory markers with no apparent source other than AEI) were not scored when the same organism was isolated from a concurrent infection at another site. The specific finding supporting each criterion, its timing after EVAR and any potential alternative source are reported for each patient in Table 1.

The ESVS 2026 guidelines, which succeed the 2020 version, comprise 81 recommendations covering definitions, diagnosis, multidisciplinary team management, antimicrobial therapy, management of intracavity and extracavity infection, and follow-up [9]. In addition to MAGIC, they recommend that [18F]FDG PET/CT or white blood cell scintigraphy combined with single-photon emission computed tomography (SPECT/CT) be considered as an adjunctive diagnostic tool (Recommendation 5, Class IIa, Level B), that white blood cell scintigraphy with SPECT/CT may be preferred within the first four months after index surgery (Recommendation 6, Class IIb, Level C), and they note that SUVmax cut-offs are unreliable [9]. Earlier classifications (Szilagyi, Samson and Bunt) were developed for surgically implanted grafts and are cited for historical context only [13,14,15].

### 2.4. Proposed Descriptive Clinical–Radiological Staging Framework

**Figure 1:**
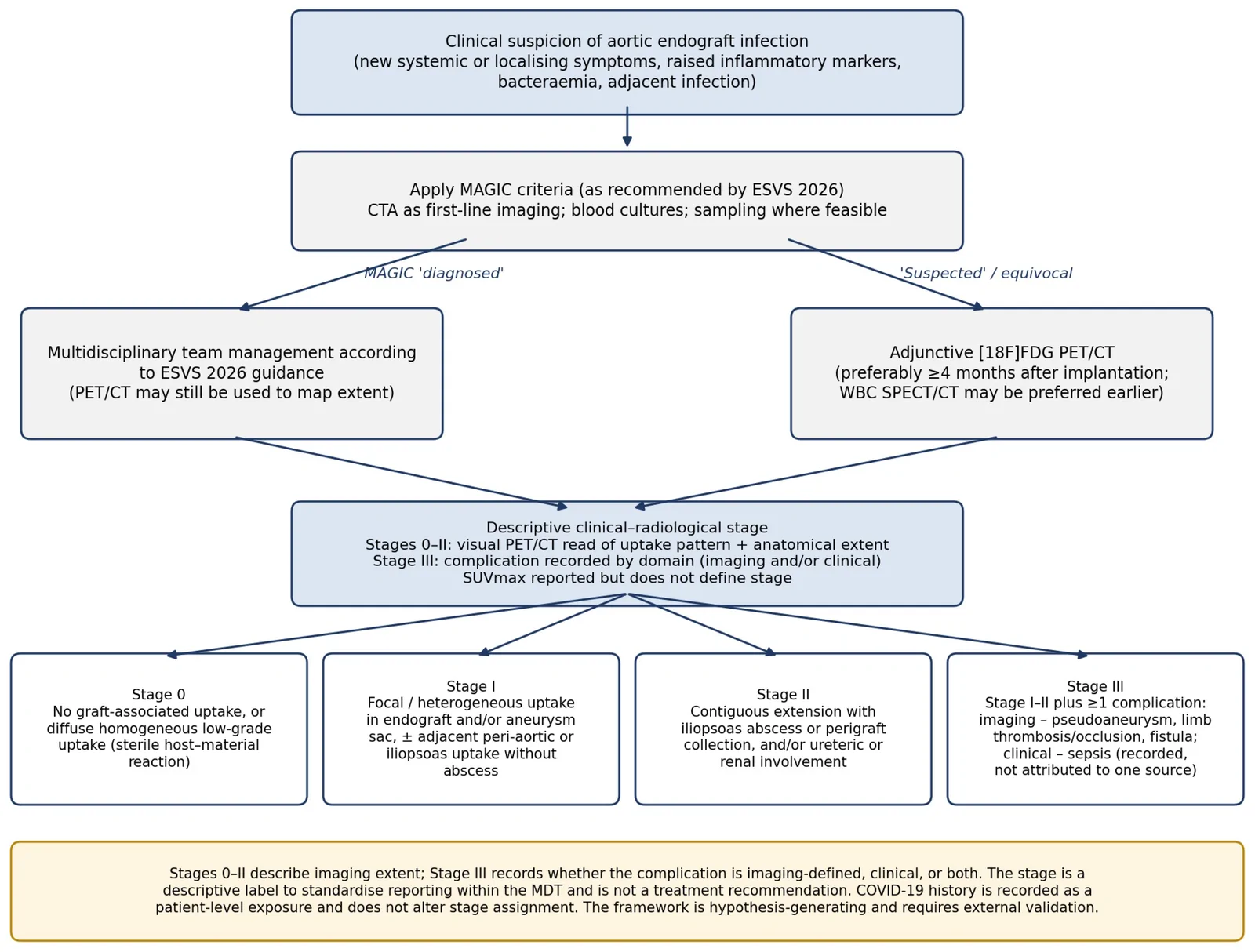
Proposed pathway for the evaluation of suspected aortic endograft infection, integrating MAGIC-based assessment (ESVS 2026) with adjunctive [18F]FDG PET/CT and the proposed descriptive clinical–radiological staging framework. Stages 0–II describe imaging extent; Stage III records imaging-defined and/or clinical complications. The stage is not a treatment recommendation. COVID-19 history is recorded as a patient characteristic and does not influence stage assignment. CTA: computed tomography angiography; ESVS: European Society for Vascular Surgery; FDG: fluorodeoxyglucose; MAGIC: Management of Aortic Graft Infection Collaboration; MDT: multidisciplinary team; PET/CT: positron emission tomography/computed tomography; SPECT: single-photon emission computed tomography; SUVmax: maximum standardised uptake value; WBC: white blood cell.

**Figure 2:**
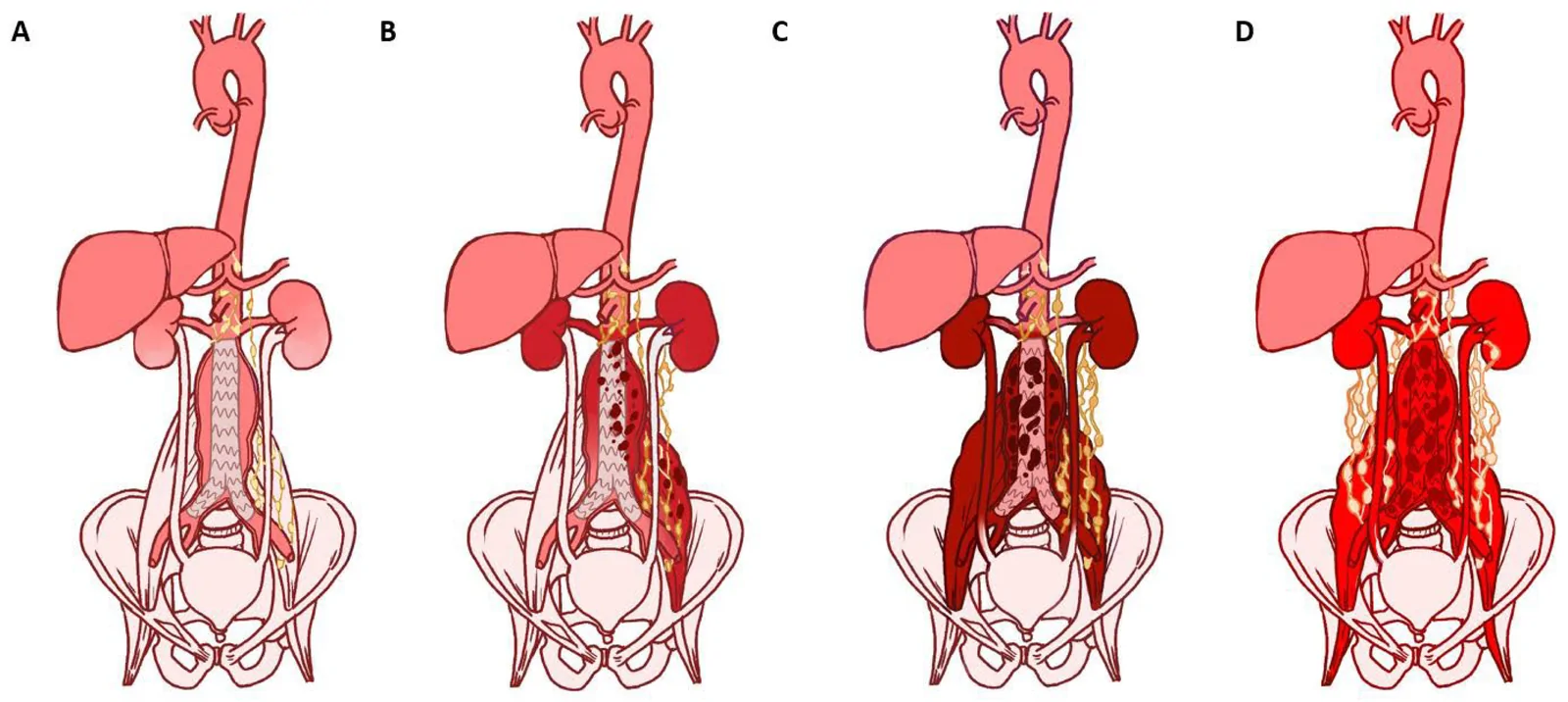
Schematic illustration of the proposed descriptive clinical–radiological stages of aortic endograft infection. (**A**) Stage 0: no graft-associated uptake, or diffuse, homogeneous low-grade uptake consistent with a sterile host–material reaction. (**B**) Stage I: focal or heterogeneous uptake in the endograft and/or aneurysm sac, with or without adjacent peri-aortic or iliopsoas uptake, without abscess. (**C**) Stage II: contiguous extension with iliopsoas abscess or perigraft collection, and/or ureteric or renal involvement. (**D**) Stage III: Stage I or II imaging findings with an imaging-defined complication (such as pseudoaneurysm, endograft limb thrombosis or fistula) and/or a clinical complication (sepsis). The illustration is descriptive and does not imply a treatment recommendation.

We propose a descriptive clinical–radiological framework for reporting the extent and complications of FDG-avid infection associated with an aortic endograft (Table 3; Figure 1 and Figure 2). Stages 0–II are defined by PET/CT uptake pattern and anatomical extent only. Stage III additionally requires a complication, which may be imaging-defined or clinical; the domain of the complication is recorded for each patient, so that imaging extent and clinical complications remain separable. SUVmax is reported descriptively and does not define stage.

- **Stage 0:** no graft-associated uptake, or diffuse, homogeneous, low-grade uptake along the endograft or aneurysm wall, consistent with a sterile host–material reaction.
- **Stage I:** focal or heterogeneous uptake in the endograft and/or aneurysm sac, with or without adjacent peri-aortic or iliopsoas uptake, without abscess or collection.
- **Stage II:** contiguous extension with an iliopsoas abscess or perigraft collection, and/or ureteric or renal involvement.
- **Stage III:** Stage I or II plus at least one complication, recorded by domain—imaging-defined (pseudoaneurysm, endograft limb thrombosis or occlusion, aorto-enteric or other fistula) and/or clinical (sepsis).

The framework is intended to be used alongside MAGIC-based diagnosis and does not replace it. It is not a diagnostic test and is not intended to guide antimicrobial or surgical treatment. Its reproducibility and clinical value require validation in independent cohorts.

**Definitionof sepsis.** The Sepsis-3 definition—life-threatening organ dysfunction caused by a dysregulated host response to infection—was used as the reference definition [16]. Because Sequential Organ Failure Assessment (SOFA) scores were not consistently recorded and could not be calculated retrospectively, sepsis was recorded as present when the referring or treating team had documented a diagnosis of sepsis at presentation, the patient required hospital admission and intravenous antimicrobial therapy, and the record contained supporting clinical and laboratory findings (Table 4). In the index patients, infection involved the endograft, the aneurysm sac and contiguous or remote sites simultaneously, so the contribution of each could not be separated. Sepsis was therefore recorded as a clinical complication present at the time of PET/CT, without attributing it exclusively to the endograft; Stage III describes a complicated presentation, not the source of sepsis.

## 3. Results

The series comprised six index cases (Group A) and five reference cases (Groups B and C); nine patients were male. Mean age was 76.0 ± 4.6 years (75.7 ± 5.0 years in Group A and 76.4 ± 4.6 years in Groups B and C). Mean pre-operative aneurysm diameter was 62.7 ± 18.5 mm (59.7 ± 20.1 mm in Group A and 66.4 ± 17.8 mm in Groups B and C). Baseline demographics are given in Table 5.

Clinical and perioperative details and outcomes are given in Table 6. No patient had post-operative fever (temperature > 38 °C). Four EVAR procedures were undertaken on an urgent basis. Seven endografts were PTFE-based (Gore Excluder^®^, W.L. Gore & Associates Inc., Newark, DE, USA) and four were polyester-based (Endurant^®^ II, Medtronic Inc., Minneapolis, MN, USA). Six patients died during follow-up: three of the six Group A patients (two infection- or aorta-related deaths and one of unknown cause) and all three Group C patients, from cancer-related causes. The median interval between EVAR and PET/CT imaging was 15 months (IQR 4–35), and the median interval from COVID-19 to the diagnosis of AEI was 14 months (IQR 10–16) (Table 1 and Table 2).

**Figure 3:**
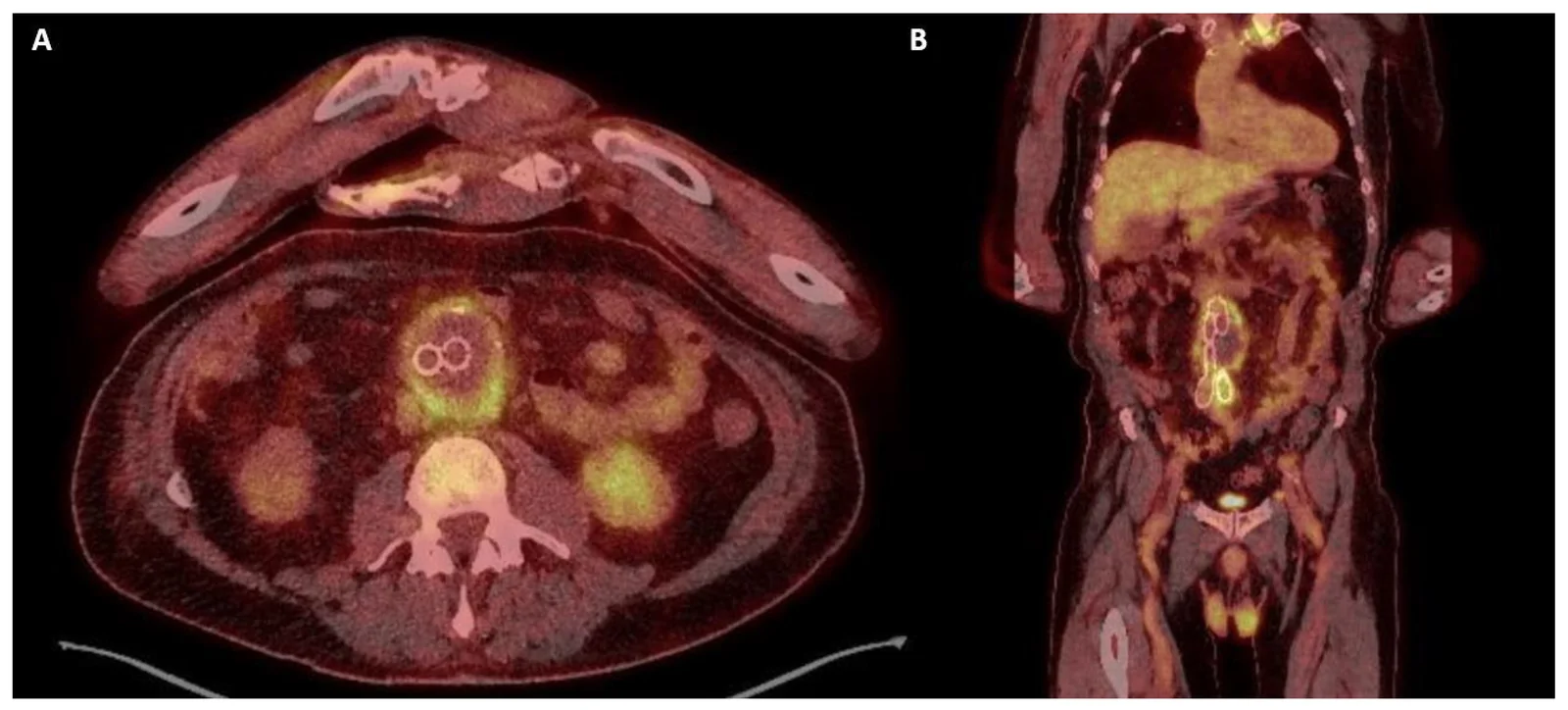
Case 1 (Group A; MAGIC suspected; Stage III—imaging extent Stage I with left iliac limb thrombosis and sepsis). (**A**): Transverse plane & (**B**): Coronal plane—showing FDG uptake in the aneurysm sac and the left iliac limb (SUVmax 6 and 16, respectively), SUVmax: maximum standardised uptake value.

**Figure 4:**
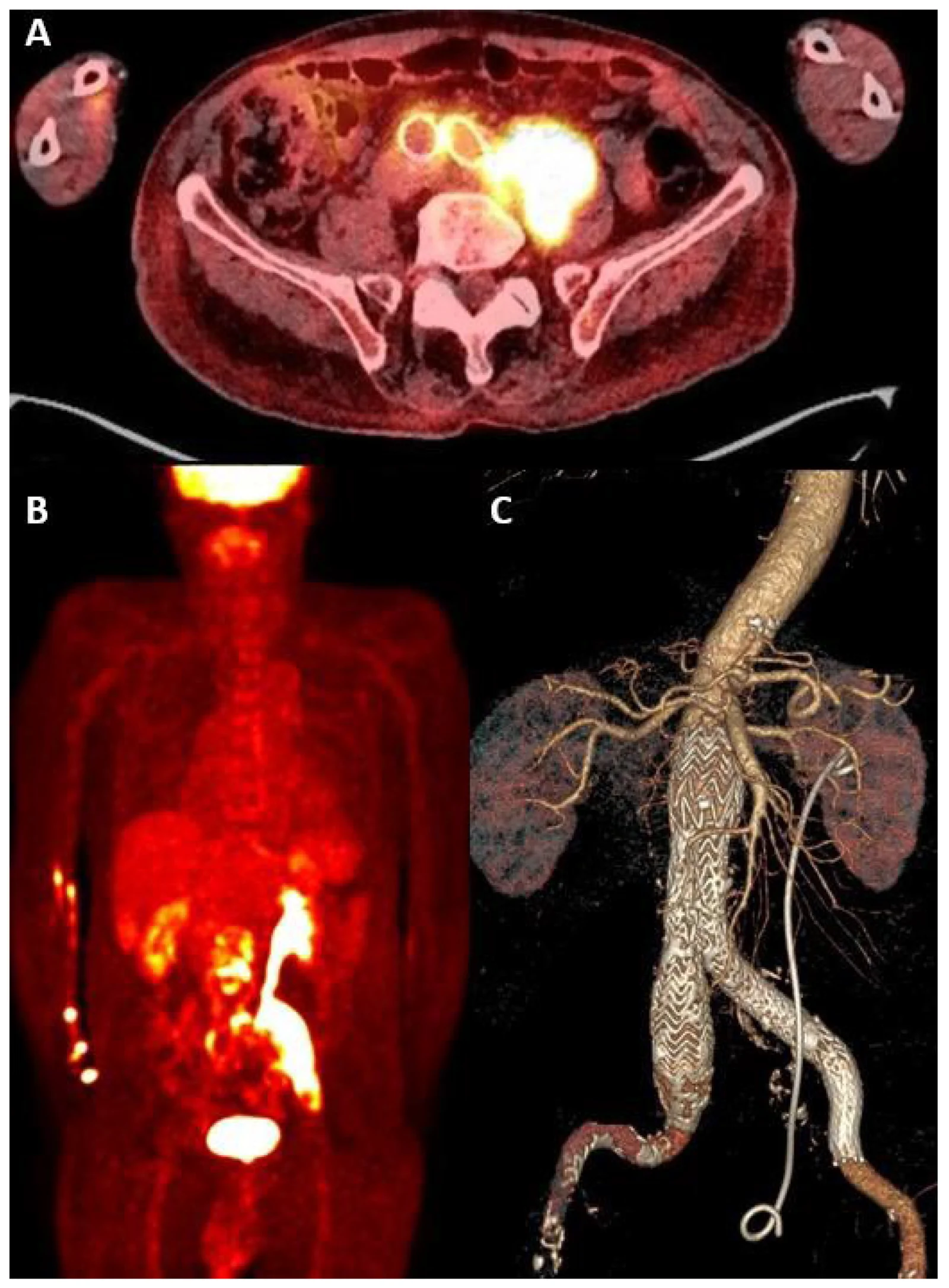
(**A**,**B**) Case 2 (Group A; MAGIC suspected; Stage III—imaging extent Stage II with sepsis) with left psoas abscess. (**A**,**B**) PET/CT FDG uptake by the EVAR graft and aortic sac with SUVmax 11 and large FDG uptake by the left psoas abscess with SUVmax 24. (**C**) CTA 3D reconstruction with a double JJ stent placed in the left ureter. EVAR: Endovascular Aneurysm Repair, PET/CT: Positron emission tomography/computed tomography, CTA: Computed Tomography Angiography, SUVmax: maximum standardised uptake value.

**Figure 5:**
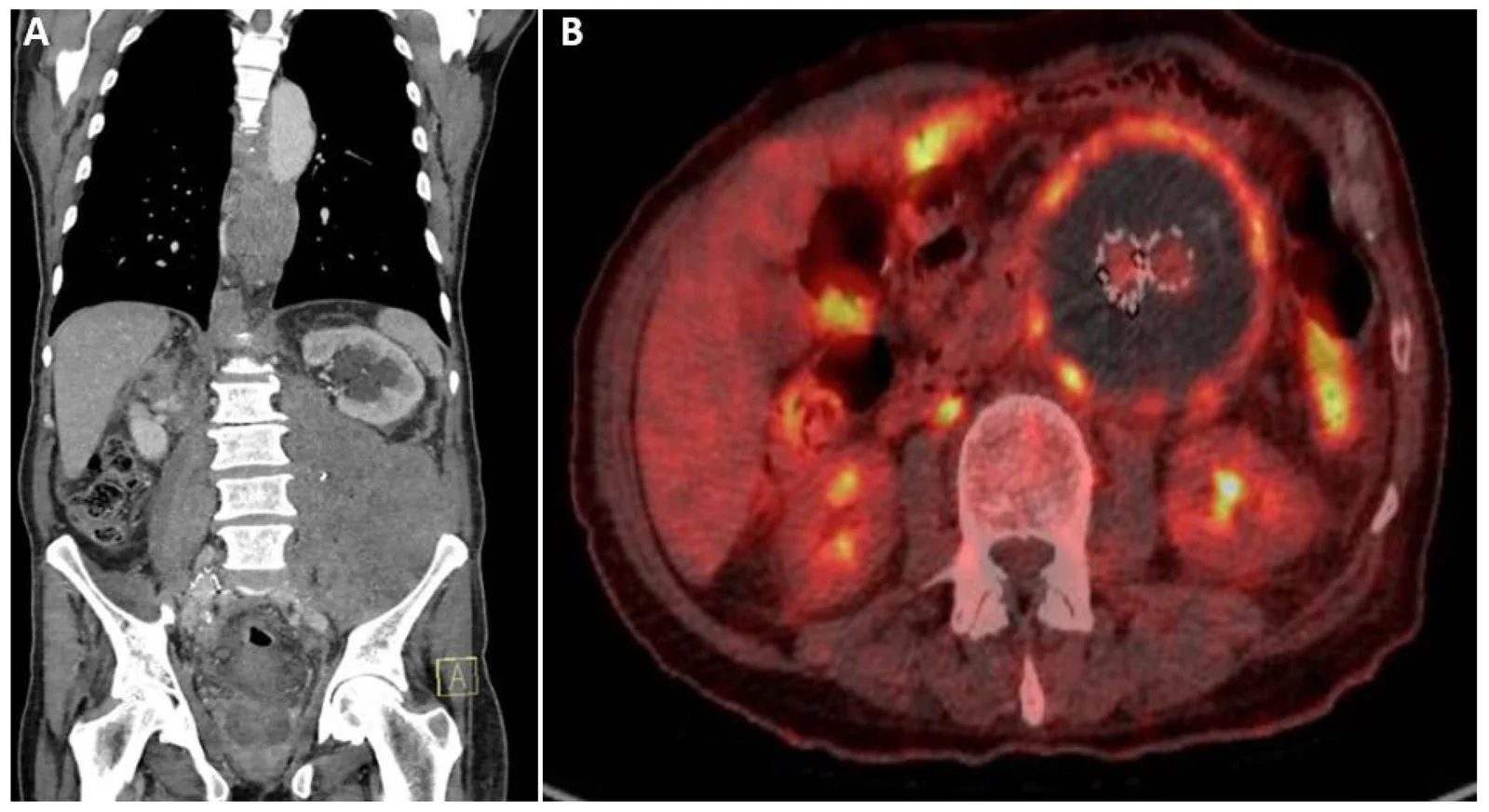
Case 5 (Group A; MAGIC suspected; Stage II) with left psoas abscess. (**A**) Left psoas abscess on CT. (**B**) PET/CT showing circumferential FDG uptake by the taortic sac with SUVmax of 6.8. PET/CT: Positron emission tomography/computed tomography, SUVmax: maximum standardised uptake value.

**Figure 6:**
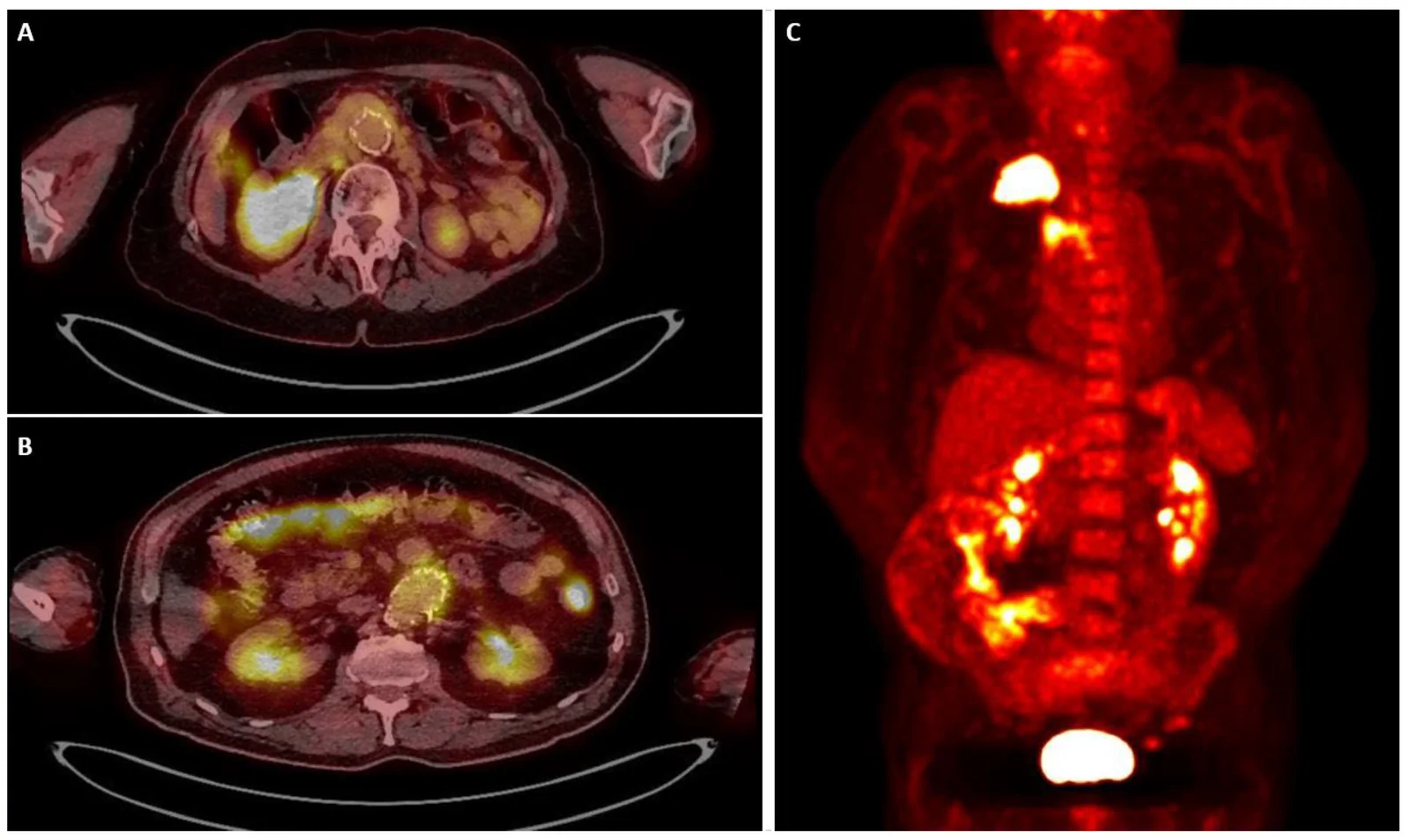
Reference cases. (**A**,**B**) Stage 0: diffuse, homogeneous low-grade uptake along theEVAR endograft and aneurysm wall in Case 7 (**A**) and Case 10 (**B**), consistent with a sterile host–material reaction. (**C**) Stage 0, Case 9: noEVAR endograft uptake;PET/CT FDG-avid lesion in the right upper lobe (SUVmax 18.9). EVAR: Endovascular Aneurysm Repair, PET/CT: Positron emission tomography/computed tomography, SUVmax: maximum standardised uptake value.

The MAGIC classification of each patient, with the finding supporting each criterion, its timing after EVAR and any potential alternative source, is shown in Table 1. One index case met the MAGIC definition of diagnosed AEI—Case 6 (*Escherichia coli* cultured from the explanted endograft, with clinical and radiological minor criteria)—and five met the definition of suspected AEI. In Case 4, the *Salmonella enteritidis* bacteraemia was not scored as a minor laboratory criterion because the same organism was isolated from a concurrent urinary tract infection, an apparent alternative source under MAGIC; Case 4 was therefore classified as suspected AEI on the basis of a single major radiological criterion (perigraft collection contiguous with the endograft on CT, 4 months after EVAR). No patient had perigraft gas on CT. All six index patients presented with fever. Five of the six index patients had an iliopsoas abscess. PET/CT findings and stages are detailed in Table 4, and representative index cases are shown in Figure 3, Figure 4 and Figure 5. Under the descriptive framework, four index endografts (Cases 1, 2, 4 and 6) were classified as Stage III. In all four, the complication included a clinical component—sepsis documented at presentation, which was the reason for tertiary referral—and in Case 1 there was also an imaging-defined complication, thrombosis of the left iliac limb of the endograft. The underlying imaging extent was Stage I in Case 1 (focal uptake in the aneurysm sac and left iliac limb, without abscess) and Stage II in Cases 2, 4 and 6 (contiguous iliopsoas abscess); the findings supporting the diagnosis of sepsis in each patient are given in Table 4. Cases 3 and 5 were classified as Stage II on the basis of contiguous iliopsoas abscess without a stage-defining complication. SUVmax of the endograft or aneurysm sac in the index cases ranged from 6.0 to 16. All five reference endografts were Stage 0: three showed no graft-associated uptake, and two (Cases 7 and 10) showed diffuse, homogeneous, low-grade uptake along the endograft consistent with a sterile host–material reaction (Figure 6). In Case 8, uptake around a femorofemoral bypass graft was reported separately and was not staged. All six index patients received parenteral antimicrobial therapy, and one (Case 6) underwent endograft explantation with axillo-bifemoral bypass (Table 4). In Case 4, the iliopsoas abscess re-accumulated during follow-up, within 203 days of PET/CT, and antimicrobial therapy was escalated (Table 2 and Table 4). Overall mortality was 54.5% (6/11): three index patients died (two infection- or aorta-related, one of unknown cause), and all three Group C patients died of malignancy.

## 4. Discussion

AEI is an uncommon yet life-threatening condition with high mortality and amputation rates [17]. Infections after endovascular repair of AAAs most commonly manifest within the first two years; the incidence of infection is similar between patients with open surgical repair (OSR) and endovascular repair [17]. However, it remains uncertain whether bacteraemia is responsible for the graft infections, or it originates from an already infected graft.

We describe six patients who developed late aortic endograft infection after documented COVID-19. Every patient also had at least one recognised alternative source of bacteraemia or cause of immunosuppression, including corticosteroids given for COVID-19 pneumonia. Because index cases were selected on both exposure and outcome, and EVAR patients with COVID-19 who did not develop infection were not studied, these observations cannot show whether COVID-19 contributes to endograft infection. Their value is descriptive: they document the clinical presentation, microbiology, PET/CT appearances and management of a rare complication during a defined period, and they identify questions that cohort studies could address.

### 4.1. Routes of Endograft Infection and Iliopsoas Involvement

The endograft is susceptible to bacterial colonisation, which can occur through various mechanisms. This includes inoculation during the index procedure, bloodstream transfer from other sites onto the fabric, exogenous interventions such as guided puncture or endovascular procedures, or infections in adjacent areas, including retroperitoneal psoas abscesses or intra-abdominal infections with fluid collections, which are prone to bacterial translocation [18,19].

Several biological mechanisms have been proposed through which SARS-CoV-2 infection, or its treatment, could in theory increase susceptibility to secondary bacterial infection, including endothelial injury, dysregulated immune responses and corticosteroid-related immunosuppression [20,21,22,23,24]. These mechanisms were not examined in our patients and remain speculative.

Iliopsoas abscess, a collection of pus within the psoas and iliacus muscles, was first described by Mynter in 1881 [25]. The susceptibility of the iliopsoas muscles to infections arises from their rich blood supply, adjacent retroperitoneal lymphatic systems, and potential trauma or haematoma within the muscle, which predisposes patients to primary abscess formation [26]. Smooth muscle haemorrhage, a notable complication of COVID-19, occurs irrespective of the type of anticoagulant administered [27]. COVID-19 and anticoagulant therapy have been associated with iliopsoas haematoma, highlighting anticoagulant use as a risk factor for this complication [28,29]. The development of bacteraemia following COVID-19 infection, especially cases with previous retroperitoneal haematoma, can lead to the formation of an iliopsoas abscess, which often delays diagnosis and effective management due to the lack of specific symptoms or signs [30,31,32,33].

In our series, Case 1 had positive blood cultures for *Staphylococcus aureus*, Case 2 had a history of cellulitis, and Case 3 had positive blood cultures for *Staphylococcus capitis* following translumbar embolization of a type II endoleak. Case 4 had a *Salmonella* urinary tract infection and *Salmonella enteritidis* bacteraemia; Case 5 had positive blood cultures for *Corynebacterium jeikeium*, *Bacteroides fragilis* and *Candida glabrata* after a CT-guided retroperitoneal lymph node biopsy and endoleak re-intervention; and Case 6 had an infected pacemaker, with *Escherichia coli* subsequently cultured from the explanted endograft. Of the reference cases, Case 7 had an *Escherichia coli* urinary tract infection without previous COVID-19, and the endograft showed only diffuse, homogeneous low-grade uptake. In Case 8, also without previous COVID-19, infection of a femorofemoral bypass graft after aorto-uni-iliac repair was suspected; no recurrent collection or new symptoms were observed during follow-up. In a retrospective analysis of patients who underwent EVAR, coil embolization of the hypogastric artery during the index procedure was associated with an increased risk of graft infection [34]. Non-typhoidal *Salmonella* bacteraemia in older adults carries a recognised risk of endovascular infection [35], so seeding of the endograft from the bacteraemia in Case 4 is biologically plausible; nevertheless, because the same organism was isolated from urine, the bacteraemia was not counted towards the MAGIC classification.

EVAR causes post-implantation syndrome, which is an acute systemic inflammatory response characterised by the presence of fever (>38 °C), leukocytosis (>12,000/mL), and increased CRP levels, all without evidence of infection. This reaction is more often described with polyester versus PTFE endografts and is associated with higher 30-day mortality and incidence of major adverse incidents. We did not find post-implantation syndrome in our reported cases.

### 4.2. AEI Imaging-PET/CT

Ultrasound can be an adjunct in the investigation of graft infection, demonstrating the presence of pseudoaneurysms and iliac limb thrombosis, which may be an early sign of infection, gas bubbles, or peri-prosthetic collections. Additionally, ultrasound can be used for guided puncture for bacteriology. However, it does not rule out infection [36]. While CTA serves as a fast and cost-effective technique, its sensitivity diminishes notably. A meta-analysis of various imaging methods for vascular graft infection concluded that CTA is insufficient as a single examination [37]. Conversely, PET-CT demonstrates superiority, particularly in early and chronic infections or those with low virulence, exhibiting higher sensitivity and specificity compared to CT alone [36,38,39,40]. Lastly, magnetic resonance angiography (MRA) has not been thoroughly evaluated for diagnosis of graft infection. MRA is not a first-line examination due to limited availability, long acquisition time and motion artefacts [41]. The ESVS 2026 guidelines recommend [18F]FDG PET/CT or white blood cell scintigraphy with SPECT/CT as adjunctive diagnostic tools in suspected vascular graft or endograft infection (Recommendation 5, Class IIa, Level B), and state that focal tracer uptake together with ancillary CT findings increases diagnostic accuracy [9].

In a study of 21 patients, Mitra et al. reported a possible cut-off SUVmax of 6.3. A comparison of FDG uptake patterns between 27 uninfected and 32 infected grafts concluded that the median SUVmax was 3.3 (interquartile range, IQR 2.0–4.2) for the uninfected and 5.7 for the infected grafts (IQR 2.2–7.8) [42]. Lastly, the FDG uptake in non-infected endografts, after EVAR, in a cohort of patients with malignancy and no signs or symptoms of infection aimed to determine the pattern of sterile or post-surgical inflammatory reaction. Sixteen oncologic patients received an Endurant^®^ stent graft. Mean values were 2.63 ± 0.48 (95% CI 2.38–2.88) and 1.90 ± 0.33 (95% CI 1.72–2.08) for SUVmax and SUV mean, respectively. The authors concluded that, even within the early postoperative period (1–4 months), mild and diffuse FDG uptake along the endograft can be a criterion for excluding infection, and [18F] FDG PET/CT can be performed immediately after EVAR [43]. This is an exciting finding, but it is essential to keep in mind that patients with malignancies may have an impaired immunological response, which could lead to differences in metabolic activity and FDG uptake.

Current ESVS guidance recommends MAGIC for diagnosis and [18F]FDG PET/CT as an adjunctive tool, notes that SUVmax cut-offs are unreliable because of overlap between infected and non-infected devices and indicates that focal uptake with ancillary CT findings improves diagnostic accuracy [9]. The proposed framework follows this approach: Stages 0–II describe uptake pattern and anatomical extent, Stage III records imaging-defined and clinical complications separately, and SUVmax is reported only as descriptive data. Its intended use is to standardise how extent is reported—for example, to distinguish uptake confined to the endograft and sac from contiguous iliopsoas abscess or urinary tract involvement—and it is not intended to diagnose infection or to guide treatment. In our series, reference endografts showed either no uptake or diffuse, homogeneous low-grade uptake, consistent with the sterile post-implantation pattern described by Lauri et al. [43]. Whether the framework is reproducible between readers and centres, and whether it carries prognostic information, must be tested in larger independent cohorts.

Iliopsoas abscess was present in five of the six index cases. The retroperitoneal compartment is anatomically vulnerable to contiguous or haematogenous spread [44], and COVID-19-associated iliopsoas haematoma and delayed iliopsoas abscess have been reported [29,32,45]. Whether this pattern reflects the patients’ other risk factors, prior COVID-19 or chance cannot be determined from this series.

PET/CT provides metabolic information that may precede structural change on CT. In our series, it distinguished the diffuse, homogeneous uptake of endografts without infection from focal uptake with contiguous extension in treated cases. FDG uptake may, however, be altered in patients with malignancy, and uptake within four months of implantation can be non-specific [43,46].

### 4.3. Treatment Approaches

In cases with AEI, the ultimate goals are to prevent aortic rupture and sepsis whilst maintaining arterial flow to the pelvis and lower extremities. Options include antimicrobial therapy, removal of the infected endograft and periaortic tissue, and flow restoration via in situ reconstruction or extra-anatomic bypass [1]. The choice of conduit varies depending on availability and patient factors, including autogenous vein, cryopreserved, prosthetic, biosynthetic, or antimicrobial-treated grafts [47,48]. Surgical explantation carries a high risk (even higher in non-elective cases) of complications and patient mortality (ranging from 29 to 46%), even in specialised centres [1,47,49,50,51]. Moulakakis et al. conducted a study on infected EVAR cases, with 40% mortality in the surgical approach compared to 50% in antibiotic therapy alone [52]. However, Shukuzawa et al. established the antibiotic approach as the standard of care in 80% of EVAR infections, with an early mortality rate of 16.7% [34]. Although it is logical to offer complete removal of infected endografts to achieve definitive treatment, conservative treatment, with or without adjuncts such as percutaneous drainage, can be implemented in patients unfit for open surgery [50,51,52]. The 2020 ESVS guidelines suggest the complete excision of all graft and infected tissue for fit patients (Recommendation Class I, Level B) [36]. A multidisciplinary approach to decision-making for these cases is warranted. The type of antimicrobial therapy is not standardised, and it depends on empirical broad-spectrum antibiotics (+/− antifungal) or culture and sensitivity results. Long-term antibiotics should be administered if the endograft was completely or partially removed or lifelong for cases treated conservatively to prevent recurrence. Along with the patient’s comorbidities, impaired immune status (malignancy, transplantation, autoimmunity, viral infections such as COVID-19) should also be taken into consideration.

Even though mortality rates are high, both with graft removal and conservative treatment with reinfection rates between 3.4% and 36%, antibiotic management might be helpful as initial management [53]. A meta-analysis by Shirasu et al. concluded that recurrent infection was more frequent after EVAR than after OSR despite similar one-year survival rates [54].

Depending on the type of antibiotic used to target the infection, outpatient parenteral antimicrobial therapy (OPAT) is a feasible solution for patients requiring long-term or lifelong treatment. Allen et al. described favourable results in their small cohort, with a 55% reduction in the length of hospital stay and 8 out of 11 patients alive after a 36-month medical follow-up [55]. Similarly, placement of a peripherally inserted central catheter (PICC) line and long-term continuation of antibiotic treatment on an outpatient basis, if feasible, was our preferred treatment strategy.

Management in our series was decided by the multidisciplinary team and is reported descriptively (Table 4); it should not be read as a recommendation. Treatment of AEI should follow current ESVS guidance, which states that graft preservation surgery may be considered in selected patients with abdominal endograft infection when surgical explantation is not feasible (Recommendation 57, Class IIb, Level C), and that long-term or lifelong antimicrobial therapy should be considered after surgical or conservative treatment when residual infection is likely (Recommendation 27, Class IIa, Level C) [9].

### 4.4. Strengths and Limitations

This study has important limitations. First, it is a small, retrospective, single-centre case series in which index cases were selected on both COVID-19 status and infection, and EVAR patients with COVID-19 who did not develop infection were not studied; the design therefore cannot estimate risk or test any association between COVID-19 and endograft infection. Second, every index patient had alternative sources of bacteraemia or causes of immunosuppression, including corticosteroid therapy. Third, the reference cases were neither matched nor randomly selected, were few, and included patients with malignancy whose FDG uptake may differ; they describe uptake in endografts without infection and cannot serve as controls. Fourth, only one of the six index cases met the MAGIC definition of diagnosed infection; the other five were classified as suspected, largely because operative or image-guided perigraft specimens were not obtained and, in Case 4, because strict application of the MAGIC requirement for no apparent alternative source excluded the bacteraemia criterion. Fifth, PET/CT studies were reviewed by vascular interventionalists rather than nuclear medicine physicians, without a formal blinding protocol, and target-to-background ratios were not measured. Although all studies were acquired on a single Philips Gemini TF system under one institutional protocol, patient-level injected activities, uptake times and blood glucose values, the study-level reconstruction settings (iterations, subsets, matrix and voxel size, post-reconstruction filter), CT exposure settings, and whether non-attenuation-corrected images—recommended to exclude attenuation-correction artefacts from metallic stent components [46]—were reviewed could not be retrieved from the retrospective records; SUVmax values should therefore not be compared directly with values obtained on other systems or with other reconstruction settings [56]. Sixth, PET/CT was performed at varying intervals after EVAR, including one study (Case 9) within the four-month window in which post-implantation uptake can cause false-positive findings, and two at approximately four months [46]. Moreover, the timing of diagnosis relative to PET/CT was also unavailable. Seventh, sepsis was identified from the clinical diagnosis documented by the treating teams rather than from prospectively recorded SOFA scores, and because infection involved the endograft and contiguous or remote sites simultaneously, sepsis could not be attributed to the endograft alone; Stage III therefore describes a complicated clinical–radiological presentation rather than the source of sepsis. Furthermore, sepsis was a clinician-documented diagnosis and could not be retrospectively verified against Sepsis-3 criteria. Finally, the proposed clinical–radiological framework was derived from these 11 patients and has not been validated; its reproducibility, diagnostic performance and clinical usefulness are unknown. The strengths of the series are complete clinical and microbiological follow-up, MAGIC classification from source records with the supporting finding reported for each criterion and independent review of every PET/CT study by two experienced readers.

Given the rarity of AEI, multicentre collaboration will be required to determine whether prior COVID-19 influences the risk of endograft infection and to test the reproducibility and clinical value of the proposed clinical–radiological framework.

Although AEI is rare, the number of patients living with endografts continues to increase with the widespread use of EVAR for AAA, and clinicians should remain alert to late endograft infection in patients presenting with unexplained systemic or localising symptoms.

## 5. Conclusions

Aortic endograft infection is a rare but life-threatening complication of EVAR. We describe six patients who developed late endograft infection, frequently with iliopsoas involvement, after documented COVID-19 and in the presence of other recognised risk factors. This series does not permit any conclusion as to whether COVID-19 contributes to endograft infection. We also propose a descriptive clinical–radiological PET/CT framework to standardise the reporting of disease extent and complications alongside MAGIC-based diagnosis; it requires validation in larger, independent, preferably multicentre cohorts before any diagnostic or therapeutic use.

## Figures and Tables

**Table 2:** Individual timeline of the index cases (Group A).

Case	EVAR Status	EVAR to AEI Diagnosis/PET/CT (Months)	Follow-Up After PET/CT (Days)	Subsequent Course
Case 1	Urgent	14	342	Alive; long-term antimicrobial therapy (lifelong oral doxycycline)
Case 2	Elective	67	89	Died (infection/aorta-related)
Case 3	Elective	20	1445	Alive; long-term antimicrobial therapy (lifelong oral doxycycline)
Case 4	Urgent	4	203	Psoas abscess re-accumulated within 203 days of PET/CT; therapy escalated (iv meropenem for 6 weeks, then lifelong amoxicillin-clavulanic acid); alive
Case 5	Elective	9	44	Died (cause unknown)
Case 6	Elective	24	23	Endograft explantation and axillo-bifemoral bypass; died (infection/aorta-related)

All index patients had PCR-confirmed COVID-19 before the diagnosis of AEI; the median interval from COVID-19 to the diagnosis of AEI was 14 months (IQR 10–16). The median interval from EVAR to PET/CT imaging for the whole series was 15 months (IQR 4–35). Follow-up is from PET/CT to last contact or death. AEI: aortic endograft infection; EVAR: endovascular aneurysm repair; IQR: interquartile range; PET/CT: positron emission tomography/computed tomography.

**Table 3:** Proposed descriptive clinical–radiological staging framework for aortic endograft infection.

Stage	Definition (Stages 0–II: PET/CT Uptake Pattern and Anatomical Extent; Stage III: Additional Complication)	Descriptive Interpretation
0	No graft-associated uptake, or diffuse, homogeneous, low-grade uptake along the endograft or aneurysm wall	Consistent with a sterile host–material reaction; infection not supported
I	Focal or heterogeneous uptake in the endograft and/or aneurysm sac, with or without adjacent peri-aortic or iliopsoas uptake, without abscess or collection	Localised process; interpret with MAGIC criteria
II	Contiguous extension with iliopsoas abscess or perigraft collection, and/or ureteric or renal involvement	Locally extensive process
III	Stage I or II plus at least one complication, recorded by domain: imaging-defined (pseudoaneurysm, endograft limb thrombosis or occlusion, aorto-enteric or other fistula) and/or clinical (sepsis)	Complicated presentation; complication domain (imaging, clinical or both) recorded

SUVmax values are reported descriptively (Table 4) and do not define stage. Sepsis is recorded as a clinical complication present at the time of PET/CT, without attribution to a single source (see Section 2). The framework is proposed for future validation; it is not a diagnostic test or a treatment recommendation. MAGIC: Management of Aortic Graft Infection Collaboration; PET/CT: positron emission tomography/computed tomography; SUVmax: maximum standardised uptake value.

**Table 4:** PET/CT findings, descriptive stage and management delivered to each patient (descriptive record; not a treatment recommendation).

Group	PET/CT	Psoas Abscess (on CT)	Descriptive PET/CT Stage	Management Delivered
**A**				
1	FDG uptake by aortic sac and left iliac stent with SUVmax 6 and 16, respectively	No	III *	PICC line, 3 months iv Flucloxacillin, Rifampicin and fluconazole followed by lifelong oral doxycycline
2	FDG uptake by the EVAR graft and aortic sac with SUVmax 11 and left psoas abscess with SUVmax 24	Yes	III *	Ureteroscopy, left double JJ stent placement, PICC line, iv daptomycin and ciprofloxacin for 3 months and re-evaluate
3	Diffuse FDG uptake within the aneurysm sac (SUVmax 6), with iliopsoas abscess on CT (drained)	Yes	II	Psoas abscess drain, PICC line, iv daptomycin for 6 weeks followed by lifelong oral Doxycycline
4	Intense FDG uptake by the aortic sac, SUVmax of 12.4; FDG-avid left psoas abscess with SUVmax of 20.1	Yes	III *	PICC line, iv ceftriaxone for 6 weeks followed by long-term oral cephalexin; on re-accumulation of the psoas abscess during follow-up (within 203 days of PET/CT), switched to iv meropenem for 6 weeks followed by lifelong amoxicillin-clavulanic acid
5	Circumferential FDG uptake by the aortic sac (SUVmax 6.8), with left psoas abscess.	Yes	II	PICC line, 1 dose of Dalbavancin, Fluconazole for 2 weeks, Metronidazole for 8 weeks followed by prescribed lifelong oral Amoxicillin- Clavulanic acid
6	Marked FDG uptake on aortic stent and iliac limbs, SUVmax 10, and a left psoas abscess, SUVmax 11	Yes	III *	Explantation of the graft with subsequent axillo-bifemoral bypass; explanted endograft culture: *Escherichia coli*
**B**				
7	Diffuse, homogeneous low-grade uptake along the endograft, with no focal endograft uptake; FDG-avid lymph nodes in the neck and mediastinum	No	0	PICC line, ceftriaxone for 3 months, then long-term oral cephalexin, as advised by microbiology for *Escherichia coli* urinary tract infection
8	No abnormal EVAR endograft uptake. Diffuse FDG uptake around the femorofemoral bypass graft and diffuse linear uptake within the right rectus femoris muscle	No collection surrounding the bypass graft	0 †	Short-term iv ciprofloxacin during hospitalisation followed by one year of oral ciprofloxacin
**C**				
9	No endograft uptake; FDG-avid right upper lobe lesion (SUVmax 18.9) with mediastinal and hilar adenopathy	No	0	Treatment for malignancy, Follow-up for EVAR
10	Diffuse, homogeneous low-grade uptake along the endograft, with no focal endograft uptake; isolated FDG-avid bone lesion in the medial left clavicle (SUVmax 19)	No	0	Treatment for malignancy, Follow-up for EVAR
11	No endograft uptake; left upper lobe nodule (SUVmax 21.1) and right upper lobe nodule (SUVmax 7.8)	No	0	Treatment for malignancy, Follow-up for EVAR

Stages were assigned according to the descriptive framework in Table 2; Cases 3 and 5 were Stage II on the basis of iliopsoas abscess. * Stage III (Cases 1, 2, 4 and 6): Case 1—imaging extent Stage I, with an imaging-defined complication (thrombosis of the left iliac limb of the endograft) and a clinical complication (sepsis); Cases 2, 4 and 6—imaging extent Stage II, with a clinical complication (sepsis). Sepsis was documented at presentation by the referring or treating team (the reason for tertiary referral) and is recorded without attribution to a single source; supporting findings: Case 1, fever with *Staphylococcus aureus* bacteraemia; Case 2, fever and weight loss with left iliopsoas abscess and left ureteric involvement requiring ureteric stenting (blood cultures negative); Case 4, fever with *Salmonella enteritidis* bacteraemia and left iliopsoas abscess; Case 6, fever and lower back pain with left iliopsoas abscess, managed by endograft explantation (blood cultures negative; *Escherichia coli* cultured from the explanted endograft). † EVAR endograft only; the femorofemoral bypass finding is reported separately and was not staged. CT: computed tomography; EVAR: endovascular aneurysm repair; FDG: fluorodeoxyglucose; PET/CT: positron emission tomography/computed tomography; PICC: peripherally inserted central catheter; SUVmax: maximum standardised uptake value.

**Table 5:** Patient demographics.

Group	Age (Years)	Sex	Comorbidities	Current Smoker	ACE Inhibitor/ARB/Antiplatelet/Anticoagulant	Aneurysm Diameter (mm)
**Group A (index cases)**						
Case 1	66	M	HTN, DLP, PAD	No	Aspirin	62
Case 2	79	M	HTN, PAD, IHD, Afib	No	Rivaroxaban, Ramipril	40 AAA, 40 LCIA
Case 3	77	M	HTN, DM, HLP, Afib	Yes	Aspirin, Valsartan	68
Case 4	80	M	HTN, HLP, Afib	Yes	Apixaban	70
Case 5	76	M	HTN	Yes	Aspirin	86
Case 6	76	M	HTN, PAD, IHD, DLP, Multiple myeloma	Yes	Ticagrelor	32
**Group B (reference cases: suspected infection at another site)**						
Case 7	74	F	HTN, DLP, PAD	No	Clopidogrel	45
Case 8	78	F	HTN, DM, PAD, DLP	Yes	Clopidogrel, Valsartan	55
**Group C (reference cases: malignancy)**						
Case 9	78	M	HTN, DM, HLP, PAD, IHD	Yes	Aspirin	70
Case 10	70	M	HTN, HLP, IHD	No	Aspirin	92
Case 11	82	M	HTN, CKD, IHD, Afib RA, Pulmonary Fibrosis	No	Rivaroxaban, Olmesartan	70

M: male, F: Female, ACE: Angiotensin-converting enzyme inhibitors, ARB:Angiotensin-2 Receptor Blockers, HTN: Hypertension, PAD: Peripheral arterial disease, IHD: Ischemic heart disease, Afib: Atrial fibrillation, DM: Diabetes mellitus, HLP: Hyperlipidaemia, DLP: Dyslipidaemia, CKD: Chronic kidney disease, RA: Rheumatoid arthritis, AAA: Abdominal aortic aneurysm, LCIA: Left common iliac artery.

**Table 6:** Clinical and perioperative details and outcomes.

Group/Cases	Vascular Access	Procedure Status	Type of Endograft	Clinical Presentation	Blood Cultures	Documented Alternative Sources of Infection or Immunosuppression	Duration (EVAR to Diagnosis of Infection or PET/CT) (Months)	Follow-Up Post PET/CT (Days)	Mortality
**Group A (index cases):**									
Case 1	Right groin percutaneous, Left groin cut-down	Urgent	Gore Excluder	Fever, fatigue, lethargy	*Staphylococcus aureus*	Shoulder dislocation and reduction; prior COVID-19 treated with corticosteroids	14	342	No
Case 2	Percutaneous Bilateral	Elective	Gore Excluder	Fever, weight loss, left psoas abscess	Negative	Left thigh cellulitis; prior COVID-19 treated with corticosteroids	67	89	Yes *
Case 3	Percutaneous Bilateral	Elective	Gore Excluder	Fever, anorexia, abdominal pain, weight loss	*Staphylococcus capitis*	Translumbar embolisation for type II endoleak; prior COVID-19 treated with corticosteroids	20	1445	No
Case 4	Percutaneous Bilateral	Urgent	Endurant II	Fever; large psoas abscess identified during work-up for malignancy	*Salmonella enteritidis*	UTI (*Salmonella*); lung adenocarcinoma; psoas abscess (*Salmonella enteritidis*); prior COVID-19 treated with corticosteroids	4	203	No
Case 5	Percutaneous Bilateral	Elective	Endurant II	Fever, diarrhoea, psoas abscess	*Corynebacterium jeikeium*, *Bacteroides Fragilis*, *Candida glabrata*	CT-guided retroperitoneal lymph node biopsy; aortic cuff and coils for type Ia endoleak; prior COVID-19 treated with corticosteroids	9	44	Yes ^†^
Case 6	Cut-down Bilateral	Elective	Gore Excluder	Fever, weight loss, lower back pain, psoas abscess	Negative	Infected pacemaker; multiple myeloma; prior COVID-19 treated with corticosteroids	24	23	Yes *
**Group B (reference cases):**									
Case 7	Cut-down Bilateral	Urgent	Gore Excluder	Rigours, fatigue	Negative	UTI (*Escherichia coli*)	16	636	No
Case 8	Cut-down Bilateral	Elective	Endurant II	Abdominal pain, lethargy	Negative	Collection surrounding the fem-fem bypass	4	689	No
**Group C (reference cases):**									
Case 9	Percutaneous Bilateral	Elective	Gore Excluder	Lower back pain	N/A	Lung adenocarcinoma, occluded right iliac branch device	0.7	226	Yes ^#^
Case 10	Cut-down Bilateral	Urgent	Endurant II	Enlarged lymph nodes	N/A	Non- Hodgkin’s lymphoma	78	607	Yes ^#^
Case 11	Percutaneous Bilateral	Elective	Gore Excluder	Asymptomatic	N/A	Lung adenocarcinoma	N/A	494	Yes ^#^

EVAR: Endovascular aneurysm repair, UTI: Urinary tract infection, PET/CT: Positron emission tomography–computed tomography, N/A: not applicable, * Infection/aortic related death, ^#^ Death from disease progression-malignancy, ^†^ Unknown.

## Data Availability

The datasets used and/or analysed during the current study are available from the corresponding author on reasonable request.

## Abbreviations

AEI	Aortic Endograft Infection
EVAR	Endovascular Aneurysm Repair
HIV	Human Immunodeficiency Virus
SARS-COV-2	Severe Acute Respiratory Syndrome Coronavirus 2
COVID-19	Coronavirus Disease 2019
CTA	Computed Tomography Angiography
UTI	Urinary Tract Infection
PET/CT	Positron Emission Tomography/Computed Tomography
FDG	Fluorodeoxyglucose
AAA	Abdominal Aortic Aneurysm
HSE	Health Service Executive
WBC	White Blood Cell
CRP	C-Reactive Protein
PTFE	Polytetrafluoroethylene
SUVmax	Maximum Standardised Uptake Value
IQR	Interquartile Range
MAGIC	Management of Aortic Graft Infection Collaboration
OSR	Open Surgical Repair
PICC	Peripherally Inserted Central Catheter
ESVS	European Society for Vascular Surgery
SPECT	Single-Photon Emission Computed Tomography
EANM	European Association of Nuclear Medicine
MDT	Multidisciplinary Team
SOFA	Sequential Organ Failure Assessment
TOF	Time-of-Flight
LYSO	Lutetium–Yttrium Oxyorthosilicate
